# Proteomic analysis of mitochondrial proteins in a mouse model of type 2 diabetes

**DOI:** 10.5830/CVJA-2010-058

**Published:** 2011-08

**Authors:** MF Essop, WA Chan, S Hattingh

**Affiliations:** Cardio-Metabolic Research Group (CMRG), Department of Physiological Sciences, Stellenbosch University, Stellenbosch, South Africa; Hatter Institute for Cardiovascular Research, Faculty of Health Sciences, University of Cape Town, South Africa; Department of Medical Physiology, Stellenbosch University Faculty of Health Sciences, Tygerberg, South Africa

**Keywords:** heart, proteomics, obesity, diabetes, contractile proteins

## Abstract

**Objective:**

Impaired mitochondrial function may contribute to the onset of contractile dysfunction with insulin resistance/type 2 diabetes. Our aim was therefore to determine alterations in the mitochondrial proteome of a mouse model of obesity/type 2 diabetes.

**Methods:**

Mitochondrial proteins were isolated from hearts collected from 18- to 20-week-old female db/db mice and compared to matched controls. We performed two-dimensional polyacrylamide gel electrophoresis to determine differentially expressed proteins. Peptides of interest were further analysed by mass spectrometry and Mascot software was employed to identify protein matches.

**Results:**

Our data showed that ATP synthase D chain, ubiquinol cytochrome-C reductase core protein 1 and electron transfer flavoprotein subunit alpha peptide levels were altered with obesity. Moreover, we found coordinate down-regulation of contractile proteins in the obese heart, i.e. α-smooth muscle actin, α-cardiac actin, myosin heavy-chain α and myosin-binding protein C.

**Conclusion:**

We propose that decreased contractile protein levels may contribute to contractile dysfunction of hearts from diabetic mice.

## Abstract

Type 2 diabetes is characterised by metabolic perturbations that may contribute to cardiac contractile dysfunction in the absence of atherosclerosis or hypertension, i.e. diabetic cardiomyopathy.^1^ For example, previous studies reported mitochondrial dysfunction in the hearts of ob/ob and db/db transgenic mice, well-described rodent models of obesity and type 2 diabetes.^2^,^3^ Here it was proposed that reduced peptide levels of mitochondrial respiratory chain complexes I, III and V in ob/ob mice, and lower expression of the F1 α-subunit of ATP synthase in db/db mice may contribute to decreased mitochondrial oxidative phosphorylation capacity. In addition, increased myocardial oxygen consumption (MVO_2_) of diabetic hearts resulted in reduced cardiac efficiency in diabetic mice, proposed to occur as a result of fatty acid-induced uncoupling of myocardial mitochondrial oxidative phosphorylation.^4^ We also recently found that obese rats displayed increased myocardial damage and attenuated respiratory capacity in response to acute oxygen deprivation.^5^

Together, these studies show that impaired mitochondrial function plays a pivotal role in the onset of contractile dysfunction associated with insulin resistance and type 2 diabetes. However, the complete identity of mitochondrial peptides involved in this process remains unclear. The aim of this study was therefore to determine alterations in the mitochondrial proteome in a transgenic mouse model of obesity and type 2 diabetes, investigating the hypothesis that db/db mouse hearts display lower expression of contractile and mitochondrial energy metabolic proteins compared to wild types.

## Methods

To investigate our hypothesis we employed 18- to 20-week-old female leptin receptor-deficient (db/db) (BKS.Cg-m+/+Lepr^db^/J strain) and heterozygous (db/+) mice. Mice were obtained from Jackson Laboratory (Bar Harbor, Maine) and exposed for one week to a reverse 12-hour light 12-hour dark cycle with free access to standard mouse chow and water. The reverse cycle was employed since mice are most metabolically active during the night period, and it allowed us to easily sacrifice mice in the middle of their night phase during our normal laboratory working hours.

All animal experiments were approved by the University of Cape Town’s Animal Research Ethics Committee (approval number 03/030) and the investigation conforms to the *Guide for the Care and Use of Laboratory Animals* published by the US National Institutes of Health (NIH Publication No. 85-23, revised 1996).

## Mitochondrial isolation procedure

On the day of the experiment, mice were anesthetised using sodium pentobarbital (50 mg/kg intra-peritoneally) and heparinised to prevent blood clotting. Mitochondria were isolated from mouse heart tissues (for both obese and age-matched lean controls) using a mitochondrial isolation kit (Sigma-Aldrich, St. Louis MO) that allows for rapid isolation of enriched mitochondrial fractions for proteomic studies.^6^,^7^ Approximately 120 mg of left ventricular tissue was dissected out from control and obese mice and weighed, cut into smaller pieces and resuspended in 10 volumes of extraction buffer A (10 mM HEPES: pH 7.5, 200 mM mannitol, 70 mM sucrose, 1 mM EGTA).

We added 0.25 mg/ml trypsin followed by incubation on ice for three minutes. After the samples were centrifuged for 15 seconds in a microfuge, the supernatant was removed by aspiration, eight volumes of extraction buffer A (containing 0.25 mg/ml trypsin) was added to the pellet and it was incubated on ice for 20 minutes. To quench proteolytic reactions, an albumin solution was added to a final concentration of 10 mg/ml. Samples were thereafter centrifuged for 15 seconds in a microfuge, the supernatant was aspirated, and the pellet was washed with eight volumes of extraction buffer A and re-centrifuged for 15 seconds. The latter step was repeated once more, where after the pellet was homogenised 20 to 30 times using a glass homogeniser.

The homogenised sample was subsequently centrifuged at 1 000 × *g* for five minutes, and the supernatant was collected and re-centrifuged at 3 500 × *g* for 10 minutes. The mitochondrial pellet was resuspended (10 mM HEPES: pH 7.4, 250 mM sucrose, 1 mM ATP, 0.08 mM ADP, 5 mM sodium succinate, 2 mM K_2_HPO_4_, 1 mM DTT) and protein concentrations were determined using the Bradford assay. We typically obtained 1 000 μg of mitochondrial proteins for our proteomic analyses.

The ReadyPrep 2D CleanUp kit (Bio-Rad, Hercules CA) was used to concentrate mitochondrial proteins by precipitation and also to wash away compounds that may have interfered with the isoelectric focusing (IEF) step. After precipitation, proteins were washed and then resuspended in an IEF/2D-compatible sample buffer [8 M urea, 2% CHAPS, 50 mM DTT, 0.2% (w/v) Bio-Lyte 3/10 ampholyte, 0.002% (w/v) bromophenol blue] and protein concentrations were determined using the RC/DC assay (Bio-Rad, Hercules CA). The complete mitochondrial isolation procedure was performed at 4°C with ice-cold solutions.

## Two-dimensional polyacrylamide gel electrophoresis (2D-PAGE)

The first dimension was performed using the PROTEAN-IEF cell (Bio-Rad, Hercules CA). Samples containing 100 μg mitochondrial protein were loaded onto 11-cm immobilised pH gradient strips (pH 5–8) (Bio-Rad, Hercules CA). Three experiments were performed for each condition (i.e. control vs obese). Strips were rehydrated under passive conditions for 12 hours at 20°C and focused at 200 V for 20 minutes. Thereafter they were linearly increased over two hours to a maximum of 8 000 V and then run (rapid ramping) to accumulate a total of 40 000 V/h. Prior to the second dimension, the immobilised pH gradient strips were first equilibrated for 15 minutes in a buffer containing 0.375 M Tris-HCL (pH 8.8), 6 M urea, 2% SDS, 20% glycerol and 2% (w/v) DTT, followed by equilibration for another 15 minutes in a buffer containing 0.375 M Tris-HCL (pH 8.8), 6 M urea, 2% SDS, 20% glycerol and 2.5% (w/v) iodoacetamide.

Subsequently, the strips were embedded in 0.5% low-melting point agarose containing 0.003% bromophenol blue (Bio-Rad, Hercules CA) on the top of Criterion XT 4–12% precast Bis Tris gels (Bio-Rad, Hercules CA), containing a 4% stacking gel. Electrophoresis was performed at 200 V; constant for 55 minutes. Gels were stained overnight with Brilliant Blue G colloidal concentrate (Sigma-Aldrich, St. Louis MO) in order to visualise the protein spots.

## Identification of mitochondrial proteins

To obtain gel images, the gels were scanned with a GS-800 calibrated densitometer (Bio-Rad, Hercules CA) using the Quantity One-4:5.2 (basic) software program (Bio-Rad, Hercules CA). The gel images were subsequently analysed using the PDQuest version 8 software program (Bio-Rad, Hercules CA) to identify differentially expressed protein spots on the gels from control and obese mice samples.

Protein spots of interest (i.e. only differentially expressed ones) were manually excised, de-stained and subjected to in-gel digestion by trypsin, followed by ElectroSpray-Injection-Quadrupole Time of Flight (ESI-Q-TOF) mass spectrometry. Data from the mass spectrometry analysis were processed and employed to create PKL files, which were used to search against the MASCOT software program (www.matrixscience.com). The latter searched the SwissProt database to identify peptide protein matches.

## Statistical analysis

Due to the small heart sizes, we pooled (for each experiment) two hearts for control and obese mice, respectively, for mitochondrial isolation. For this study we performed three independent mitochondrial isolation experiments as described above. Subsequently, three independent gels were performed per mitochondrial isolation experiment for db/db and db/+ mouse hearts, and only changes found in two or more experiments were selected for further analysis. Data are presented as increased or decreased fold change versus matched controls. Statistical differences between groups were calculated using the Student’s *t*-test. Statistical significance was considered when *p* < 0.05.

## Results

**Fig. 1.**
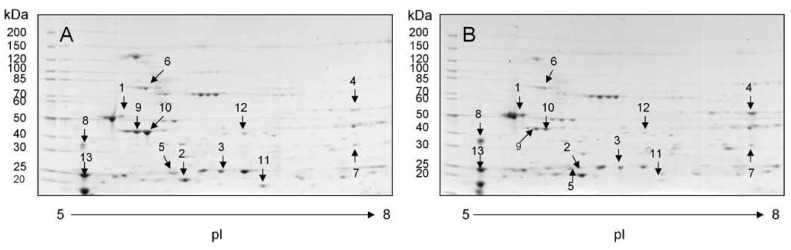
Two-dimensional PAGE patterns of mitochondrial proteins. Heart mitochondria were isolated from normal and obese female mice and purified from interfering substances with the first-dimension separation. Proteins were separated by two-dimensional PAGE and detected by Coomassie staining. The spots containing proteins that were subsequently identified are numbered. A: control and B: obese. Molecular weight is given in kDa.

Two-dimensional PAGE analysis highlighted several differences between obese db/db mouse hearts versus matched db/+ controls (Fig. 1). Proteins of interest were further analysed by mass spectrometry and peptides were identified using software as described in the methods section. We divided altered mitochondrial protein expression into two broad groupings, i.e. regulating mitochondrial energy metabolism (Table 1), and forming part of the myocardial contractile apparatus (Table 2).

**Table 1.** Proteins Identified In Obese Female Mice: Mitochondrial Energy Metabolism

*Spot*	*SSP*	*Mr (Da)*	*pI*	*Mascot score*	*Protein name*	*Swiss prot accession number*	*% Sequence coverage*	*No of peptides*	*Fold increase or decrease*	*Functional group or function*
1	2305	56265	5.19	873	ATP synthase subunit beta,	P56480	44	18	2.7	ATP synthesis, (catalytic subunit)
2*	5002	18664	5.52	273	ATP synthase D chain	Q9DCX2	70	11	2.5	ATP synthesis, (rotational mechanism)
3*	6003	53420	5.75	280	Ubiquinol cytochrome-C reductase core protein 1	Q9CZ13	21	7	–2.1	Electron transport
4	8406	59830	9.22	693	ATP synthase subunit alpha	P56757	30	15	14.9	ATP synthesis (regulatory subunit)
5	3101	27640	5.19	297	NADH dehydrogenase flavoprotein 2	Q9D6J6	45	9	–1.8	Electron transfer
6	2903	80724	5.51	563	NADH ubiquinone oxidoreductase 75 kDa subunit	Q91VD9	26	18	–2	Electron transfer
7*	9302	35360	8.62	623	Electron transfer flavoprotein subunit alpha	Q99LC5	51	15	5.9	Electron transfer

Data represent differentially expressed proteins between control and obese female mice. Only proteins that were significantly different are reported (*p* < 0.05). The SSP number is assigned by the PDQuest software program (Bio-Rad, Hercules CA). *Proteins identified in two or more independent experiments.

**Table 2.** Proteins Identified In Obese Females: Contraction/Cytoskeletal

*Spot*	*SSP*	*Mr (Da)*	*pI*	*Mascot score*	*Protein name*	*Swiss prot accession number*	*% Sequence coverage*	*No of peptides*	*Fold increase or decrease*	*Functional group or function*
8	0203	32718	4.69	429	Tropomyosin 1, alpha chain	P58771	34	11	2.5	Striated muscle contraction
9*	3201	42381	5.23	268	Actin, smooth muscle	P62737	22	7	–2.3	Structural constituent of cytoskeleton
10*	3203	42334	5.23	318	Actin, alpha cardiac	P68033	35	10	–4.0	Structural constituent of cytoskeleton
11*	7002	224349	5.57	225	Myosin heavy chain	QO2566	2	6	–3.8	Muscle contraction
12*	5401	141799	6.06	179	Myosin binding protein C, cardiac	O70468	6	9	–1.9	Muscle contraction
13	0206	22390	5.03	283	Myosin light chain	P05977	33	8	1.7	Muscle contraction

Data represent differentially expressed proteins between control and obese female mice. Only proteins that were significantly different are reported (*p* < 0.05). The SSP number is assigned by the PDQuest software program (Bio-Rad). *Proteins identified in two or more independent experiments.

Seven proteins regulating mitochondrial energy metabolism were differentially expressed (statistically significant) between obese and control hearts. These data show both up- and down-regulation of key regulators of electron transfer in the electron transport chain (ETC) and mitochondrial ATP production. However, expression of only three proteins, i.e. ATP synthase D chain (increased 2.5-fold), ubiquinol cytochrome-C reductase core protein 1 (decreased 2.1-fold) and electron transfer flavoprotein subunit alpha (increased 5.9-fold) were altered in at least two independent experiments.

Interestingly, we also found differentiated expression for several contractile/cytoskeletal proteins (Table 2), although only four displayed decreased expression in at least two independent experiments. Here α-smooth muscle actin, α-cardiac actin, myosin heavy-chain (MHC) α and myosin-binding protein C (MyBP-C) protein levels were coordinately down-regulated in obese versus control hearts.

## Discussion

We previously found a significant increase in body weight and fasting blood glucose levels for 18- to 20-week-old db/db female mice versus matched controls.^8^ For the current study, 2D-PAGE illustrated differences between control and obese hearts for female mice that we broadly categorised into two groups, i.e. related to energy metabolism and contraction/cytoskeleton. Here we identified changes in several proteins that play a role in mitochondrial energy metabolism, although only three, i.e. ATP synthase D chain, ubiquinol cytochrome-C reductase core protein 1 and electron transfer flavoprotein subunit alpha were identified as real changes.

ATP synthase D chain peptide levels were up-regulated for the db/db mouse versus controls. ATP synthase D chain is a non-enzymatic component of the F_0_ channel of the F_1_F_0_ ATP synthase that links the flow of protons (from within the inter-mitochondrial membrane space) through its F_0_ channel to ATP synthesis that occurs on F_1_. We propose that this may represent an adaptive mechanism by the obese female heart to increase proton transfer and thereby enhance mitochondrial ATP production. In agreement with this notion, we found a marked induction of electron transfer flavoprotein subunit alpha. The latter usually serves as an electron acceptor for dehydrogenases, and plays a role in the transfer of electrons along the mitochondrial ETC.

Conversely, we found a decrease in peptide levels of ubiquinol cytochrome-C reductase core protein 1, a subunit of complex III of the electron transfer chain that catalyses transfer of electrons from coenzyme Q to cytochrome C. These data suggest that despite attempts by hearts from the obese animals to augment mitochondrial ATP production, decreased ubiquinol cytochrome-C reductase core protein 1 peptide levels likely contribute to impaired mitochondrial ATP production. In agreement, we previously found diminished cardiac respiratory capacity in female db/db mice compared to matched controls.^8^ It is likely that ATP synthase D chain and electron transfer flavoprotein subunit alpha may also be down-regulated with prolonged persistence of the diabetic phenotype.

We also found coordinated down-regulation of key contractile/cytoskeletal proteins in the obese heart, i.e. α-smooth muscle actin, α-cardiac actin, MHCα and MyBP-C. Together these peptides play a crucial role in ensuring sustained myocardial contractile function and cytoskeletal support.^9^ The contractile proteins identified in this study were associated with the isolated mitochondrial fraction and likely represent proteins closely associated with interfibrillar mitochondria. These data are consistent with previous work that found an MHC isoform switch during the onset of diabetes, i.e. decreased MHCα and increased MHCβ expression.^10^ MyBP-C is a thick filament-associated protein and provides an additional regulatory step to myocardial contraction.^11^ MyBP-C gene mutations can cause hypertrophic cardiomyopathy, 11 while its absence (cMyBP-C null mice) significantly attenuates *in vivo* left ventricular function.^12^

Together our data are in agreement with an earlier study that found increased contractile dysfunction with older db/db female mice (aged 16–18 weeks).^13^ We therefore propose that it is likely that lower contractile protein expression may indeed contribute to impaired contractile function observed in the diabetic heart. However, additional studies are required to confirm these changes within the cytosolic fraction of hearts from diabetic animals.

## Conclusion

This study found that diabetic mouse hearts displayed altered expression of mitochondrial metabolic peptides together with the coordinated down-regulation of several cardiac contractile/cytoskeletal proteins. We propose that attenuated contractile protein expression may contribute to the onset of diabetic cardiomyopathy.

## References

[1] Boudina S, Abel ED (2007). Diabetic cardiomyopathy revisited.. Circulation.

[2] Boudina S, Sena S, O’Neill BT, Tathireddy P, Young ME (2005). Reduced mitochondrial oxidative capacity and increased mitochondrial uncoupling impair myocardial energetics in obesity.. Circulation.

[3] Boudina S, Sena S, Theobald H, Sheng X, Wright JJ (2007). Mitochondrial energetics in the heart in obesity-related diabetes: direct evidence for increased uncoupled respiration and activation of uncoupling proteins.. Diabetes.

[4] How OJ, Aasum E, Severson DL, Chan WY, Essop MF (2006). Increased myocardial oxygen consumption reduces cardiac efficiency in diabetic mice.. Diabetes.

[5] Essop MF, Anna Chan WY, Valle A, Garcia-Palmer FJ, Du Toit EF (2009). Impaired contractile function and mitochondrial respiratory capacity in response to oxygen deprivation in a rat model of pre-diabetes.. Acta Physiol.

[6] Lopez MF, Kristal BS, Chernokalskaya E, Lazarev A, Shestopalov AI (2000). High-throughput profiling of the mitochondrial proteome using affinity fractionation and automation.. Electrophoresis.

[7] Rabilloud T, Kieffer S, Procaccio V, Louwagie M, Courchesne PL (1998). Two-dimensional electrophoresis of human placental mitochondria and protein identification by mass spectrometry: toward a human mitochondrial proteome.. Electrophoresis.

[8] Essop MF, Chan WY, Taegtmeyer H (2007). Metabolic gene switching in the murine female heart parallels enhanced mitochondrial respiratory function in response to oxidative stress.. FEBS J.

[9] Walker CA, Spinale FG (1999). The structure and function of the cardiac myocytes: a review of fundamental concepts.. J Thorac Cardiovasc Surg.

[10] Paulson DJ, Gupta M, Zak R, Zhao J (1992). Effects of exercise training and diabetes on cardiac myosin heavy chain composition.. Mol Cell Biochem.

[11] Flashman E, Redwood C, Moolman-Smook J, Watkins H (2004). Cardiac myosin binding protein C: its role in Physiology and disease.. Circ Res.

[12] Brickson S, Fitzsimons DP, Pereira L, Hacker T, Valdivia H (2007). *In vivo* left ventricular functional capacity is compromised in cMyBP-C null mice.. Am J Physiol Heart Circ Physiol.

[13] Aasum E, Hafstad AD, Severson DL, Larsen TS (2003). Age-dependent changes in metabolism, contractile function, and ischemic sensitivity in hearts from db/db mice.. Diabetes.

